# Oral immunotherapy with enteric-coated capsules for allergic rhinitis caused by house dust mites

**DOI:** 10.3389/falgy.2024.1345929

**Published:** 2024-05-07

**Authors:** Han-Zhong Zhang, Wei Xie, Wen-Cheng Zhou, Jian Chen, Ying Wang, Yuan-Yuan Zhu, Ting-Huan Wen, Lei Cheng

**Affiliations:** ^1^ Department of Otorhinolaryngology, Wuxi Hospital of Traditional Chinese Medicine, Wuxi, China; ^2^ Department of Otorhinolaryngology & Clinical Allergy Center, The First Affiliated Hospital, Nanjing Medical University, Nanjing, China; ^3^ Mites Laboratory, School of Basic Medical Sciences, Fudan University, Shanghai, China; ^4^ International Centre for Allergy Research, Nanjing Medical University, Nanjing, China

**Keywords:** allergic rhinitis, mites, oral immunotherapy, subcutaneous immunotherapy, enteric-coated capsules

## Abstract

**Background:**

Oral immunotherapy (OIT) is a promising allergen-specific approach in the management of food allergy; however, studies on OIT for allergic rhinitis (AR) have rarely been reported. The purpose of this study is to evaluate the efficacy and safety of OIT using enteric-coated capsules for AR induced by house dust mites.

**Methods:**

A total of 49 patients with AR were enrolled, including 25 who received subcutaneous immunotherapy (SCIT) and 24 who received OIT. The clinical efficacy and safety in both groups were evaluated.

**Results:**

After 1 year of treatment, both SCIT and OIT demonstrated significant therapeutic effects. OIT was found to be more effective than SCIT in reducing the total AR symptom score and improving the results of nasal provocation tests. Local and systemic adverse reactions were observed in the SCIT group, while none were reported in the OIT group.

**Conclusion:**

OIT is an effective and safe treatment for mite-induced AR.

## 1 Introduction

Current options for the prevention and treatment of allergic rhinitis (AR) include allergen avoidance, pharmacotherapy (use of symptomatic medications), allergen immunotherapy (AIT) and health education (1). Unlike pharmacotherapy, which offers instant symptomatic relief, AIT is the sole disease-modifying option that has the potential to alter the natural course of allergic response and provide symptom relief even after therapy discontinuation (2). If allergens cannot be avoided, AIT serves as the only cure for immunoglobulin E (IgE)-mediated type I respiratory allergies, such as AR (3). Subcutaneous injection is the most common route in AIT. However, subcutaneous immunotherapy (SCIT) causes pain and potential severe adverse reactions, thus restricting its wide use. Sublingual immunotherapy (SLIT) is effective for suppressing the nasal and ocular symptoms of AR; however, factors have to be weighed against its less well-established efficacy and potentially higher costs (4, 5). Therefore, urgency has been refreshed to develop AIT that is safer, cheaper, less laborious, more effective, and highly compliable.

Oral administration may be an alternative route of AIT (4). SCIT enhances the suppression on the proliferation of antigen-specific Th2-cells and the progressive increase of antigen-specific IgG, while oral immunotherapy (OIT) causes supplementary changes through the mucosa and/or regional lymph nodes (6, 7). OIT can successfully desensitize allergic individuals to allergic foods, such as peanuts (8). But studies on OIT for AR induced by house dust mites (HDMs) have rarely been reported. It has shown that in administering HDMs OIT, the oral route does not seem to offer a superior efficacy over the conventional routes; this conflict may be attributed to patient selection and allergen bioavailability to the gut due to gastric enzymatic degradation (9, 10). HDMs are a prevalent indoor allergen causing allergic respiratory diseases, including AR and allergic asthma (11). The objective of this study is to verify a hypothesis that *Dermatophagoides farina* (*Df*) encapsulated in enteric-coated capsules might effectively treat HDMs-induced AR through OIT, achieving a similar or superior effect to that of SCIT. In this study, we hope to clarify the effectiveness and safety of OIT with enteric-coated capsules and provide evidence for its application against HDMs-induced AR.

## 2 Materials and methods

### 2.1 Study population

We included patients, all referring to the same hospital, with a diagnosis of moderate-to-severe AR, according to the Allergic Rhinitis and its Impact on Asthma (ARIA) guidelines (12, 13). Inclusion criteria were: age 5–60 years; AR and proven sensitization to HDMs; complaints of perennial nasal itching, sneezing, nasal congestion and running nose. The main exclusion criteria were: uncontrolled asthma, nasal polyps, respiratory infection, tuberculosis, neoplastic and autoimmune diseases. In this randomized, open-label controlled study, a total of 54 patients were selected by the researchers. The patients or their guardians signed the informed consent. Researchers assigned unique identification numbers to patients based on the order of their visits, and these identification numbers were randomized into the OIT or SCIT groups in a 1:1 ratio according to a computer-generated randomization list. The numbers were placed and opened by a colleague who was not involved in the study. The study was conducted in accordance with the Declaration of Helsinki, and the protocol was approved (No.2014050302) by the institutional review board of Wuxi Hospital of Traditional Chinese Medicine.

Out of the 54 patients with AR, a total of 49 successfully completed more than 1 year of immunotherapy; during the study period, there were dropouts observed in both treatment groups due to relocation: 3 patients from the SCIT group and 2 patients from the OIT group had to discontinue their participation as they changed their city of residences. The SCIT group included 25 patients (14 males and 11 females, ages 6–45 years, mean age 22.5 ± 11.8 years), and the OIT group included 24 patients (13 males and 11 females, ages 8–39 years, mean age 25.6 ± 8.9 years). There was no statistical significance in gender and age between two groups (*p* > 0.05). All patients were diagnosed with HDMs-induced allergy, based on a positive skin prick test (SPT) and a positive nasal provocation test (NPT) (1, 12).

### 2.2 Allergy testing

The SPT reagents were provided by ALK-Abelló A/S (Hørsholm, Denmark). Totally, 14 common inhalant allergens (10 HEP/ml) including *Df* and *Dermatophagoides pteronyssinus* (*Dp*) were chosen for SPT on the forearm. Saline was used in the negative control group, and histamine hydrochloride (10 mg/ml) in the positive control group. The SPT is carried out according to the practical guidelines (1, 12). Skin index (SI) was the ratio of the diameter of an allergen-induced wheal to that of a histamine-induced wheal. SI has four grades: +, 0.3 ≤ SI < 0.5; ++, 0.5 ≤ SI < 1.0; +++, 1.0 ≤ SI < 2.0; ++++, SI ≥ 2.0. SPT ≥ 0.5 was determined as positive. At the enrollment, all patients got positive for *Df* and *Dp*, with a SPT ≥ 0.5 (SPT ≥ 2.0 mostly). Sensitization to only HDMs was tested in 19 patients (38.8%), and the combination of other inhalants in 30 patients (61.2%).

*Df* allergen solution used in NPT and enteric-coated capsules were provided by the Mites Laboratory of Fudan University School of Basic Medical Sciences (Shanghai, China). Three concentrations were set: No. 0 (allergen protein 1 µg/ml), No.1 (allergen protein 100 µg/ml) and No. 2 (allergen protein 1,000 µg/ml). Normal saline was used as a reference solution. The patients were instructed to take a rest for 15 min in the examination room. A 0.3 cm diameter filter paper dipped in allergen solution was alternately placed on the head of the inferior turbinate in both nostrils, for 10 min per placement. If the reference solution was positive, subsequent NPT was cancelled; if the lower concentration allergen solution showed positive reaction, subsequent NPT using higher-concentration solution was cancelled. Each NPT was observed for 10 min, and the results were evaluated and rated by subjective measures before and after NPT. A point (0–3) was given to each of the four clinic symptoms, including nasal itching, sneezing, nasal congestion and running nose (0 = none, 1 = mild, 2 = moderate, or 3 = severe). An increase by ≥ 3 points in the sum of the nasal symptom scores indicated a positive NPT. If the initial nasal allergen response was negative after 10 min, then the extract concentration was increased every 15 min. The dose was increased in a step-wise fashion until a positive result was obtained, or a maximum concentration was given if no significant reaction appeared. *Df* NPT results showed that all patients were positive, and 12 patients were combined with asthma. The NPT was conducted before AIT (baseline) and 1 year after treatment.

For each patient, 2 ml of blood was sampled before (baseline level) and at 1 year after treatment initiated. Blood serum was separated from the sample. Total IgE (tIgE) was measured using enzyme-linked immunosorbent assay (ELISA) (Roche Diagnostics Ltd., Germany). *Dp* specific IgG4 (sIgG4) was determined using ELISA according to the manufacturer's instructions (ALK-Abelló A/S, Hørsholm, Denmark). In brief, sIgG4 was detected using mouse monoclonal anti-human IgG4 antibodies, followed by HRP-coupled sheep anti-mouse IgG. Colorimetric detection for ELISA was done and optical density was measured on a spectrophotometer. Plate-to-plate normalization was performed by a testing control serum pool with established antibody levels for the antigens on each plate. Unspecific binding of detection antibodies was excluded by performing buffer controls. All determinations were carried out in duplicates, and the results of each sample were shown as means (14).

### 2.3 Immunotherapy regimens

The SCIT group underwent treatment with standardized *Dp* allergen extracts (Alutard SQ, ALK-Abelló A/S, Hørsholm, Denmark) using a rush immunotherapy schedule, as detailed below: in the dosage accumulation phase after admission, three injections (daily) were administered in the first 3 days in different arms with a 30-min interval (day 1: 10, 100 and 1,000 SQ-U; day 2: 2,000, 4,000 and 6,000 SQ-U; day 3: 8,000, 10,000 and 20,000 SQ-U), and two injections were administered in the next 2 days (day 4: 30,000 and 40,000 SQ-U; day 5: 50,000 and 50,000 SQ-U). At day 6, one injection of 100,000 SQ-U (9.8 μg of major allergens) was given. Later, the dosage per injection reached the maintenance level. At day 7, the patients were discharged. At day 28 after discharge, the dosage in one injection was maintained at 100,000 SQ-U, and the patient was observed for at least 30 min after injection. Afterward, a dose of 100,000 SQ-U was injected every 6 weeks. Once reactions were found at injection site and across the body, the dosage was adjusted according to product specifications.

OIT group received oral intake of *Df* enteric-coated capsules, dissolving in the small intestine after passing through the stomach intactly. The protein content was 1 mg/ml in the solution, *Der f* 1 protein content was 90 μg/ml, and *Der f* 2 protein content was 12 μg/ml. The empty enteric-coated capsules were manufactured by Anhui Huangshan Capsule Co., Ltd. (Anhui, China). Each capsule contained dust mite allergen and 0.1 g of matrix flour. The capsules were divided into two types: No. I containing 3 µg of *Df* and No. II containing 48 µg of *Df*, both prepared in the laboratory of our hospital. The treatment was accomplished within two stages: up-dosing phase and maintenance phase. During the first 5 days, a cascade of dosages of 3 µg, 6 µg, 12 µg, 24 µg and 48 µg were given. Thereafter, daily 48 µg was maintained for 2 months; then, the patients took the capsule every other day for 10 months. The patient took capsules on an empty stomach at home, and meals were allowed 30 min later. Within 8 weeks after the initiation of immunotherapy, the patient was allowed to take H1-antihistamines or intranasal glucocorticoids depending on the severity of nasal symptoms. Drug use was recorded every month.

### 2.4 Efficacy assessment

The efficacy was assessed by comparing the total nasal symptoms score (TNSS) (15) and a visual analogue scale (VAS) (16) before and 1 year after treatment initiation. Four symptoms were scored, including nasal congestion, nasal itching, sneezing and running nose. Each symptom was scored with a point of 0–3 (0: asymptomatic; 1: mild; 2: moderate; 3: severe). The patient used the VAS to score each nasal symptom as 0–10 points: 0 indicating no distress and 10 indicating the most serious symptom. The scores of each symptom were summed as the total nasal VAS score. During the assessment, the drug use was divided into frequent use (one per week), occasional use (once per month) and never use.

### 2.5 Safety assessment

In the SCIT group, we looked for local adverse reactions, including swelling (or itching and pain) at the injection site, as well as systemic adverse reactions. In the OIT group, local adverse reactions referred to gastrointestinal reactions; in this group we looked for systemic adverse reactions as well.

### 2.6 Statistical analysis

SPSS 20.0 software (IBM SPSS Statistics, United States) was used for analyzing the TNSS, VAS score, serum tIgE, sIgG4, and NPT index score. Measurement data in normal distribution were labeled as mean ± SD. A paired *t*-test was used for the comparison within the group; independent *t-*test sampling was used for comparison between groups. The measurement data not in normal distribution were expressed as median and 25th and 75th percentiles. Wilcoxon rank sum test was adopted for the comparison within the group, and Mann-Whitney *U* test for comparison between groups. The chi-square test was used for counting data. The statistical significance was defined as *p* < 0.05.

## 3 Results

### 3.1 Symptom scores

As shown in Table 1, the TNSS was 5.12 ± 3.26 in the SCIT group and 3.50 ± 2.17 in the OIT group after 1 year of immunotherapy, both significantly lower than those at baseline (8.96 ± 1.86 and 9.33 ± 1.90, respectively) (*p* < 0.001, both). As shown in Table 2, the decrease in the TNSS after treatment was more important in the OIT group (5.83 ± 2.58), when compared to the SCIT group (3.84 ± 2.53) (*p* = 0.009).

**Table 1:** Total nasal symptom score and total nasal VAS score in the SCIT and OIT groups before and after treatment (mean ± SD).

Group	n	TNSS				VAS			
		Baseline	After 1 year	*t*	*p*	Baseline	After 1 year	*t*	*p*
SCIT	25	8.96 ± 1.86	5.12 ± 3.26	7.60	<0.001	21.16 ± 7.26	12.48 ± 7.84	5.66	<0.001
OIT	24	9.33 ± 1.90	3.50 ± 2.17	11.07	<0.001	23.29 ± 6.53	10.00 ± 4.54	10.68	<0.001
MD (95% CI)		0.37 (−0.71, 1.46)	−1.62 (−3.21, −0.03)			2.13 (−1.84, 6.11)	−2.48 (−6.17, 1.21)		
*t*		0.695	−2.057			1.079	−1.361		
p		0.491	0.046			0.286	0.181		

CI, confidence interval; MD, mean difference; OIT, oral immunotherapy; SCIT, subcutaneous immunotherapy; SD, standard deviation; TNSS, total nasal symptom score; VAS, visual analogue scale.

**Table 2:** Comparison of change in total nasal symptom score and total nasal VAS score in the SCIT and OIT groups before and after treatment (mean ± SD).

Variable	*n*	SCIT	*n*	OIT	*t*	*p*
TNSS differences	25	3.84 ± 2.53	24	5.83 ± 2.58	2.73	0.009
VAS differences	25	8.68 ± 7.67	24	13.29 ± 6.10	2.32	0.025

OIT, oral immunotherapy; SCIT, subcutaneous immunotherapy; SD, standard deviation; TNSS, total nasal symptom score; VAS, visual analogue scale.

### 3.2 VAS scores

As shown in Table 1, the total nasal VAS score was 12.48 ± 7.84 in the SCIT group and 10.00 ± 4.54 in the OIT group after 1 year of immunotherapy, both significantly lower than those at baseline (21.16 ± 7.26 and 23.29 ± 6.53, respectively) (*p* < 0.001, both). As shown in Table 2, the decrease in the total nasal VAS score after treatment was more important in the OIT group (13.29 ± 6.10), when compared to the SCIT group (8.68 ± 7.67) (*p* = 0.025).

### 3.3 Symptomatic medication usage

The percentages of patients who did not receive symptomatic medications were 8.0% (2/25) and 4.2% (1/24) before the treatment, and significantly increased to 72.0% (18/25) and 87.5% (21/24) after 1 year treatment in the SCIT group and the OIT group, respectively (*p* < 0.001, both); however, there was no significant difference between two groups (*p* > 0.05).

### 3.4 NPT results

The NPT data from 21 patients in the SCIT group and 23 patients in the OIT group were analyzed. As shown in Table 3, the concentration of *Df* was 643 µg in the SCIT group and 596 µg in the OIT group, and significantly higher than those at baseline (510 µg and 187 µg, respectively). The number of patients with high concentration *Df* increased in the SCIT group, but without statistically significant difference (*p* = 0.65). Interestingly, the number of patients with high concentration *Df* also increased in the OIT group, with significant difference (*p* = 0.008). There was no significant difference in the concentration distribution between two groups after 1 year of treatment (*p* > 0.05).

**Table 3:** Numbers of positive NPT patients in the SCIT and OIT groups before and after treatment.

Group		NPT concentration (µg/ml)						*χ* ^2^	*p*
		1		100		1,000			
		No.	%	No.	%	No.	%		
SCIT	Baseline	4	19.0	7	33.3	10	47.6	0.87	0.65
	After 1 year	3	14.3	5	23.8	13	61.9		
OIT	Baseline	7	30.4	13	56.5	3	13.0	9.65	0.008
	After 1 year	3	13.0	7	30.4	13	56.5		

NPT, nasal provocation test; OIT, oral immunotherapy; SCIT, subcutaneous immunotherapy.

After 1 year of immunotherapy, the score of the NPT was reduced in 10 patients (47.6%) from the SCIT group, and in 18 (78.3%) in the OIT group, with a significant difference between the two groups (*p* = 0.035).

### 3.5 Serological results

As shown in Table 4, compared to that at baseline level, the serum tIgE level did not change in the SCIT group (*p* > 0.05), and increased in the OIT group, but without significant difference after 1 year of immunotherapy (*p* > 0.05). There was no significant difference in serum tIgE levels between the two groups before and after treatment (*p* > 0.05, both).

**Table 4:** Serum total IgE changes in the SCIT and OIT groups before and after treatment [median (IQR)].

Group	*n*	Total IgE (IU/ml)		*Z*	*p*
		Baseline	After 1 year		
SCIT	25	197.00 (98.50; 624.00)	197.00 (97.62; 797.50)	−1.63	0.10
OIT	24	227.50 (141.50; 465.25)	249.50 (96.00; 440.00)	−0.37	0.71
*Z*		−0.66	−0.60		
*p*		0.51	0.95		

IgE, immunoglobulin E; IQR, interquartile range; OIT, oral immunotherapy; SCIT, subcutaneous immunotherapy.

At the same time, serum *Dp* sIgG4 was tested in 22 patients from the SCIT group and 19 patients from the OIT group before and after treatment. As shown in Table 5, compared to the baseline level, the serum *Dp* sIgG4 level significantly increased in the SCIT group (*p* < 0.001) and decreased in the OIT group, but without significant difference (*p* > 0.05) after 1 year of immunotherapy. There was no significant difference in serum *Dp* sIgG4 level between the two groups before the treatment (*p* > 0.05), but after 1 year treatment, the *Dp* sIgG4 level in the SCIT group was significantly higher than that in the OIT group (*p* < 0.001).

**Table 5:** Serum *Dp* specific IgG4 changes in the SCIT and OIT groups before and after treatment [median (IQR)].

Group	*n*	*Dp* specific IgG4 (AU/ml)		*Z*	*p*
		Baseline	After 1 year		
SCIT	22	153.50 (71.75; 435.00)	5,497.00 (2,516.75; 20,583.75)	−4.07	<0.001
OIT	19	213.00 (123.00; 362.00)	146.00 (84.00; 390.00)	−1.17	0.24
*Z*		−0.59	−4.84		
*p*		0.56	< 0.001		

*Dp, Dermatophagoides pteronyssinus*; IgG*,* immunoglobulin G; IQR*,* interquartile range; OIT*,* oral immunotherapy; SCIT*,* subcutaneous immunotherapy.

### 3.6 Safety

Non-severe local adverse reactions occurred in all patients in the SCIT group, with self-resolution, without requiring any treatment. Systemic adverse reactions developed within 30 min after the injection in three patients (a total of 9 times) during the dosage accumulation phase, but they resolved immediately after injection of dexamethasone and/or epinephrine. No local or systemic adverse events reported in the OIT group.

## 4 Discussion

OIT, an emerging route of immunotherapy, can effectively desensitize patients with persistent IgE-mediated allergy caused by cow's milk, hen's egg, and peanuts (17). However, its efficacy and safety in the treatment of AR remains widely unassessed. A study has shown that short-term OIT is effective for cedar pollinosis in a Japanese population (18). Our previous single blind placebo-controlled study has also confirmed that OIT based on enteric-coated capsules is effective for AR caused by *Df* (19). Enteric-coated capsules are solid dosage forms, which are designed to bypass the stomach and release the drug in the small intestine, and protect the integrity of the *Df* from the acid in the stomach. It may delay the onset of drug effect and increase the duration of action. This formulation is also characterized with advantages of longer dosing intervals, high convenience and compliance of patients (20). In this open-label, randomized control trial, SCIT and OIT achieved significant therapeutic effects in AR patients, as evidenced by lower nasal symptom scores and less drug use than those before treatment. In addition, OIT was more effective than SCIT in reducing TNSS and VAS scores, indicating that OIT may be an alternative AR treatment option.

SCIT has demonstrated efficacy in reducing allergic respiratory symptoms and been widely used. However, the higher risk of systemic reactions or life-threatening anaphylaxis, as well as the inconvenience in administration, have limited the application of SCIT. Additionally, injection-caused pain may elicit resistance to SCIT in certain pediatric patients. A recent study has reported high safety and efficiency of OIT in elderly patients with allergic rhinoconjunctivitis and asthma due to grass pollen (7). In our study, OIT with enteric-coated capsules also showed stronger clinical efficacy and safety than SCIT. One might expect that OIT capsules would be more convenient for patients to take in at home regularly, compared to in-hospital administration of SCIT. Considering these, self-administered OIT has been shown to be an effective and safe alternative.

AIT has shown capability in relieving allergic symptoms, as well as increasing nasal tolerance to allergens. NPT is considered as a gold standard for assessing clinical relevance in patients sensitized to *Dermatophagoides* species and identifying the allergens before commencing AIT (21–23). It has been confirmed that the responsiveness of NPT correlates with the severity of AR in *Dermatophagoides* species-sensitized patients (24). In our study, NPT results showed favorable changes that are consistent with those of symptoms in both groups after immunotherapy. The NPT improvement rate in the OIT group was significantly higher than that in the SCIT group, indicating that OIT is more advantageous in nasal desensitization.

AIT may change the serum level of antibodies. It has shown that long-term OIT brings about obvious immunological changes (25). In the present study, serum tIgE level did not change significantly before and after immunotherapy in both groups. Studies have reported heterogeneity (increase or decrease) and homogeneity in tIgE response during AIT (26). This controversy may arise from the difference in study period and sampling methodology.

Allergy is defined as an immune response induced by the interaction between the antigen and IgE antibodies bound to mast cells and basophils that induce the release of inflammatory mediators that cause the clinical symptoms (27). sIgG4 provide an anti-inflammatory effect and may competitively bind to Fcɛ receptors of mast cells and basophils, thus blocking the activation and degranulation of effector cells (25). The increase of sIgG4 is correlated with the reduction of basophil activation during AIT (28). So, serum IgG4 may suppress the allergen-triggered release of basophil histamine and the binding of IgE-allergen complexes to B cells. Many studies have shown that the increase in sIgG4 level is significantly associated with the alleviation in nasal symptoms during AIT (29–31). In our study, the level of *Dp* sIgG4 in the SCIT group increased significantly, which is consistent with the improvement of symptoms. However, no significant change in *Dp* sIgG4 level was observed in the OIT group after treatment. The positive correlation between allergen sIgG4 and clinical outcomes has been reported in some but not all studies (32). One study has reported that sIgG4 may peak at any time during AIT period (33). Another has reported that sIgG4 increases in a time-dependent fashion, then declines by 90% (26). As for OIT, oral intake of transgenic rice containing 7 linked major T-cell epitopes from Japanese cedar pollen allergens effectively suppresses allergen-specific T-cell proliferation, but does not change serum sIgG4 (34). In addition, in the peptide immunotherapy, oral feeding to mice of transgenic rice seeds expressing the T cell epitope peptides before systemic challenge inhibited the development of allergen-specific serum IgE and IgG antibody and CD4^+^ T cell proliferative responses (35). So, immunoreactive IgG4 has an obscure correlation with clinical response. One possible explanation for this discrepancy is that AIT may induce the production of new sIgG4 with augmented binding specificity and/or affinity (29). This may explain why no significant change in *Dp* sIgG4 levels was observed during OIT in our study. The mechanism of AIT is very complex. Enteric capsules can better stabilize antigens without inducing the destructive effects of stomach acid. Except causing changes in serum sIgG4, macrophages and epithelial cells may be important targets for immunotherapy to suppress allergen-induced Th2 responses (36).

In our study, all patients in the SCIT group developed mild local adverse reactions within 30 min after injection. Additionally, three patients experienced a total of nine systemic adverse reactions that were promptly resolved with symptomatic treatment. These findings emphasize the importance of monitoring patients for at least 30 min following SCIT injections. However, during the dosing build-up phase of rush immunotherapy, 3 patients (12%) in the SCIT group experienced systemic adverse reactions that appeared to be more severe compared to those in the conventional SCIT. Indeed, accelerated immunotherapy may be associated with an increased risk of systemic adverse reactions. Literature reports indicate that the incidence of systemic adverse reactions during rush immunotherapy ranges from 27% to 100% (37). It's worth noting that no local or systemic adverse events were observed in the OIT group, indicating that OIT presents a safer treatment option for AR.

SCIT carries a risk of systemic reactions, including rare fatal anaphylaxis, whereas OIT typically causes localized reactions but rare systemic reactions. Although no severe systemic side-effects were reported, OIT did induce some gastrointestinal adverse effects, because the allergens were mostly given in their native form (18). The most commonly observed symptoms are mild-to-moderate, typically represented by oropharyngeal pruritus or transient abdominal pain that is either self-limited or easily treated with antihistamines (38). Due to the fact that OIT patients take medications at home and all adverse events were self-reported, the incidence of which might be influenced by the patient's ability to recognize adverse events related to OIT. Hence, it remains a challenge to accurately assess the nature and number of adverse events, especially during at-home treatment. For example, gastrointestinal adverse events are particularly difficult to be defined, because they may arise in many clinical scenarios during OIT (39).

The present study has some limitations. First, we only observed the effect of OIT on dust mite sensitized patients, but did not further explore the relevant mechanisms and carry out serum allergen-specific IgE test. Second, the patients included in this study were from a single medical center and the sample size was small, which may increase the risk of selection bias. Therefore, more samples from multiple centers and mechanistic exploration are required to evaluate the efficacy of OIT with *Df* enteric-coated capsules in patients with AR and provide more evidence for the clinical application of OIT in the future. Furthermore, additional investigations are needed to address specific knowledge gaps in optimal dose, duration, age of initiation, maintenance schedule, cost-effectiveness, predictors of risk and therapeutic response, thus maximizing the efficacy and minimizing the risks of OIT.

In summary, both SCIT and OIT demonstrated significant therapeutic effects after 1 year of treatment in this study. However, OIT exhibited superior efficacy and safety to SCIT. The *Df* enteric-coated capsules appear to be a convenient, effective, and safe option for patients with mite-induced AR. Future real-world studies are required to elucidate the effectiveness and safety of OIT in AR patients.

## Data Availability

The original contributions presented in the study are included in the article/Supplementary Material, further inquiries can be directed to the corresponding author.
